# The impact of meteorological parameters on the scrub typhus incidence in Baoshan City, western Yunnan, China

**DOI:** 10.3389/fpubh.2024.1384308

**Published:** 2024-04-24

**Authors:** Yun-Yan Luo, Alan Frederick Geater, Jia-Xiang Yin

**Affiliations:** ^1^School of Public Health, Dali University, Dali, Yunnan, China; ^2^Department of Epidemiology, Faculty of Medicine, Prince of Songkla University, Hat Yai, Songkhla, Thailand

**Keywords:** distributed lag non-linear model, meteorological parameters, scrub typhus incidence, high-known endemic areas, lag pattern

## Abstract

**Background:**

Scrub typhus has become widespread across various regions in China in recent decades, causing a considerable burden on residents. While meteorological variables significantly impact the spread of scrub typhus, there is insufficient quantitative evidence illustrating this association in known high-endemic areas.

**Methods:**

A distributed lag non-linear model was applied to explore the relationship between meteorological parameters and scrub typhus incidence from 2010 to 2019 in Baoshan City, western Yunnan Province, China.

**Results:**

High monthly mean (20°C) and maximum (30°C) temperatures were associated with a peak risk of scrub typhus in the current month. Higher minimum temperatures and higher relative humidity were followed by increasing cumulative risks over the ensuing 3 months. Higher precipitation was followed by increasing cumulative risk over the ensuing 2-month period, peaking at around 30 cm.

**Conclusion:**

The non-linear lag associations between meteorological parameters and scrub typhus incidence suggest that higher monthly minimum temperature and relative humidity could be associated with an increased risk of scrub typhus in the subsequent several months, while warm temperature is more likely to impact the occurrence of scrub typhus in the current month.

## 1 Introduction

Scrub typhus (ST) is a re-emerging vector-borne disease caused by the bacterium *Orientia tsutsugamushi* (*Ot*). Mites are the main host and vector. Small mammals play a crucial role in the transmission of *Ot* in natural environments, infected mites could be carried to non-endemic areas. Humans are the occasional host when bitten by infected mites (1, 2). Currently, scrub typhus transmission has extended beyond the traditional distribution areas, with reported cases in Chile and Dubai (2). Moreover, misdiagnosis and underreporting of the disease frequently occur due to inadequate diagnosis assay and a lack of awareness among health care workers (3). Furthermore, an effective licensed vaccine is not yet available, and antibiotic resistance in the treatment of scrub typhus remains uncertain (4). Without prompt and appropriate treatment, the disease can result in severe symptoms and multiple organ failure, with a 70% mortality rate (5). Scrub typhus has thus become a growing health concern worldwide, with an increasing number of reports and inadequate medical resources. To date, potentially effective preventive measures (including rodent control, insecticide use, and human intervention) and surveillance systems for scrub typhus have been performed by a few countries, such as China, South Korea, Japan and Thailand, etc. (3, 6–8). In China, scrub typhus was first documented in 1948, and the Chinese National Notifiable Infectious Disease Reporting Information System has listed it as a common infectious disease starting in 2006 (2). Yunnan Province is one of the scrub typhus natural foci in China. From 2010 to 2019, 41,323 cases of scrub typhus were reported in Yunnan Province, with 56.10% originating from western Yunnan. Of these, 9,034 cases were reported in Baoshan City, 8,254 in Lincang city, and the remaining cases in Dehong prefecture. Previous studies have exhibited a high clustering of scrub typhus cases in Yunnan Province based on the spatiotemporal analysis, with Longling City (belonging to Baoshan City) having the highest number of cases reported in the recent decade (9–11). So as to mitigate the burden and threat of scrub typhus for local residents and travelers, it is essential to clearly understand the high occurrence mechanism of scrub typhus in western Yunnan.

Previous studies indicated that scrub typhus exhibited varying seasonal patterns in different regions. Korea illustrated high outbreaks of scrub typhus between October to November (12). In India, the occurrence of scrub typhus spans from July to February during the monsoon and post-monsoon seasons (13). Nepal reached a peak of scrub typhus during August and September, while in Thailand the scrub typhus reached a peak from June to November (8, 14). In China, a summer type of scrub typhus was observed in the south of the Yangtze River (i.e., Guangdong, Sichuan and Yunnan Province). Notably, recent years in Yunnan Province have shown a second peak in September and October. The seasonal pattern of scrub typhus strongly suggests that meteorology could have an important impact on the spread of scrub typhus, most likely through its effect on the life-cycle of mites and the survival and reproduction of small mammals. Recent studies have indicated that temperature, relative humidity and precipitation are the limiting factors in controlling the spread of scrub typhus in China (15–22). These studies indicated: (1) High temperature and precipitation are positively associated with the abundance and reproduction of mites, indirectly increasing the probability of mites transmitting *Ot* from nature to humans. However, the impact conditions of temperature and precipitation vary across geographic regions. (2) Humidity is a determining factor reported by previous studies; only an appropriate range of humidity facilitates the abundance of mites and host small mammals, indirectly influencing the transmission of scrub typhus in nature. (3) Temperature and precipitation also influence human activity. Warmer temperatures without precipitation tend to attract people to engage in outdoor activities, thereby increasing the risk of infection by scrub typhus from nature. Consequently, these meteorological parameters that exhibit geographic variations should be investigated further to develop a comprehensive disease profile that enhances understanding of the occurrence and spread mechanism of scrub typhus in high endemic areas. Despite its importance, few studies have conducted quantitative analyses linking meteorological parameters to the incidence of scrub typhus in southwestern China, especially in the high-occurrence Yunnan Province. Clarifying this association in high-endemic areas can offer scientific insights for local governments, aiding proactive measures and reducing human health burden. Elaborating on the impact of climate variables on the incidence of scrub typhus can reveal the early warning conditions for the spread of scrub typhus and provide valuable scientific evidence applicable to regions with similar climates and environments.

Yunnan Province spans seven climate types. Baoshan City mainly features a tropical and subtropical climate, providing appropriate habitats and food sources for the survival and development of small mammals and mites. In the past decades, the numbers of cases of scrub typhus were the highest in Baoshan City among all cities in Yunnan Province. This environment poses a potential transmission condition for scrub typhus and other vector-borne disease. One study showed that in recent years, apart from *Leptotrombidium deliense,* which traditionally is the vector of scrub typhus in the whole Yunnan Province, some other vector species such as *L. scutellare*, *L. rubellum* and *L.sialkotense* also coexist in Yunnan Province (23). These species are commonly found in other provinces of China, but recent reports indicate their successive presence in Yunnan Province. This could potentially be attributed to climate change, indirectly accelerating the occurrence of scrub typhus, which suggests the need for further research on this issue. Therefore, this study aimed to explore the effect of six meteorological parameters on the incidence of scrub typhus based on the distributed lag non-linear model from 2010 to 2019 in Baoshan City, western Yunnan Province. The lagged associations identified should facilitate quantifying the risk period for the spread and transmission of scrub typhus and offer valuable insights for the relevant government authorities to control the spread of scrub typhus in humans.

## 2 Materials and methods

### 2.1 Study setting

**Figure 1:**
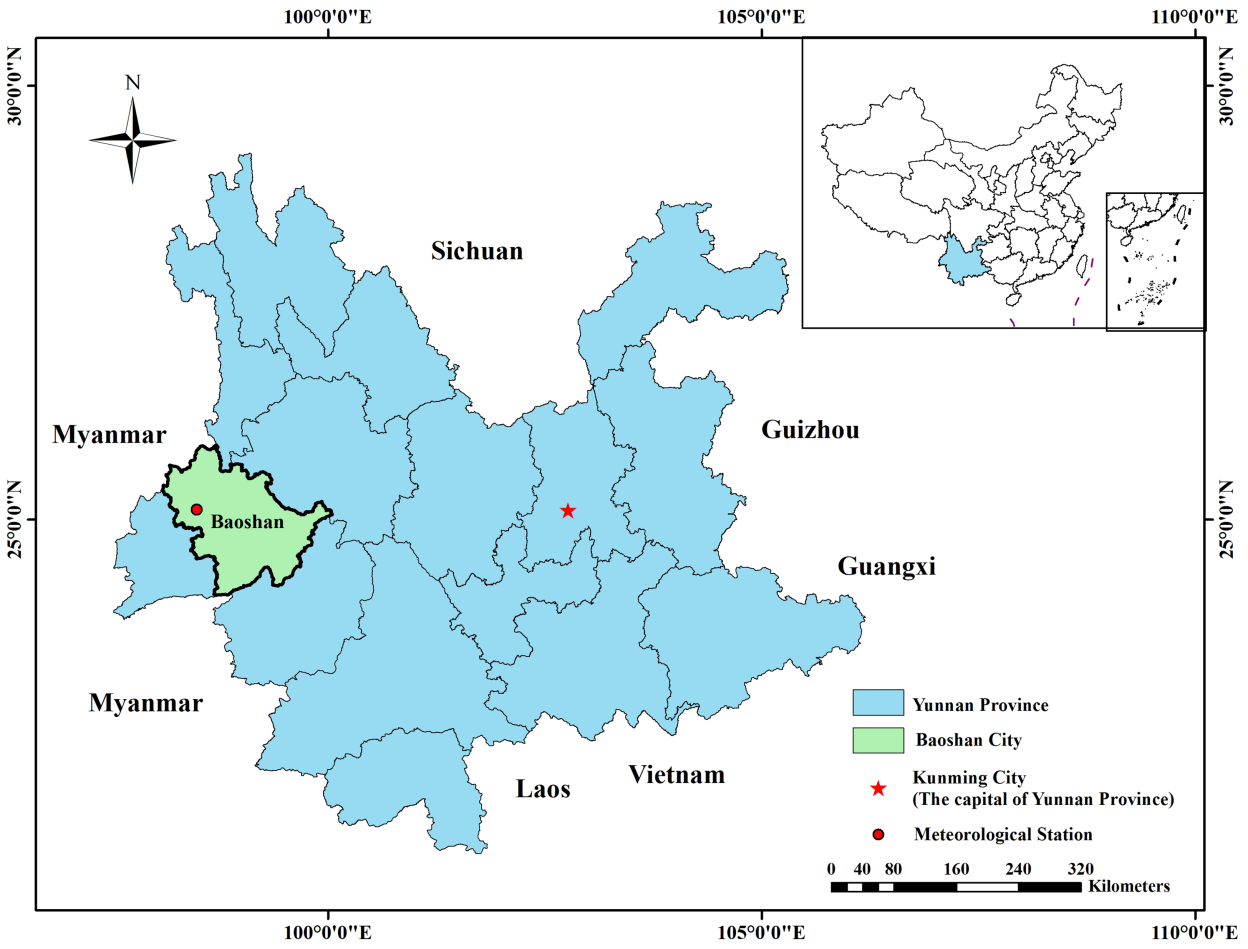
Geographic location of Baoshan City in western Yunnan Province, China. The map was created in ArcGIS 10.2 using the political boundaries from the National Geomatics Center of China for illustrated sample location only.

Baoshan City (E 98°25′ to 100°02′, N 24°08′ to 25°51′), located in western Yunnan Province under the south part of Hengduan mountain range (Figure 1), has been considered the main scrub typhus natural focus with the highest number of scrub typhus cases at city level in Yunnan Province. This city’s altitude varies from 535 to 3,781 meters, and the significant difference in altitude leads to complex climate types and abundant vegetation. From 2010 to 2019, the average population was about 2,574,400 (including 5 counties). Furthermore, Baoshan City holds profound cultural heritage, attracts numerous tourists annually, and serves as a vital international commercial route connecting the China and Myanmar border. Therefore, the occurrence of scrub typhus outbreak in local area may lead to increased probability of infections in both residents and travelers and a more widespread transmission.

### 2.2 Data collection

#### 2.2.1 Scrub typhus cases and incidence

The annual and monthly number of scrub typhus cases in Baoshan City from 2010 to 2019 were obtained from the Chinese National Notifiable Infectious Disease Reporting Information System in the Chinese Center for Disease Control and Prevention (China CDC).^1^ Diagnoses for all cases were conducted by clinicians adhering to standardized diagnostic criteria according to the technical guidelines for preventing and controlling scrub typhus (2009).^2^ Medical institutions are obligated to submit the report of scrub typhus cases within 24 h in a unified format through the web-based surveillance system. Probable cases are those with a history of visiting in scrub typhus endemic areas or field activities within 3 weeks before the onset of illness with clinical manifestations such as fever, lymphadenopathy and rash. Clinically confirmed cases are probable cases accompanied by the eschar. Laboratory confirmed cases are the probable or clinically confirmed cases meeting any of the following positive laboratory tests: (1) Weill-Felix test; (2) Indirect immunofluorescence antibody assay; (3) PCR test; (4) Isolation of *Orientia tsutsugamushi*. Probable cases necessitate further confirmation. All obtained cases in this study were both clinically confirmed cases or laboratory confirmed cases.

Population census data in Baoshan City from 2010 to 2019 were retrieved from the National Bureau of Statistics of China and the Statistics Yearbook of Yunnan Province.^3^

#### 2.2.2 Meteorological parameters data

This study focused on monthly mean, maximum and minimum temperatures (°C), monthly temperature range (°C), and monthly means of relative humidity (%) and precipitation (cm) in Baoshan City from 1st January 2010 to 31st December 2019 with data provided by the China Meteorological Data Service Center.^4^ Monthly temperature range refers to the difference between the maximum and minimum temperature recorded in a specific location during a particular month. This measure provides insights into the temperature variability within a given period.

### 2.3 Statistical analysis

#### 2.3.1 Monthly distribution of meteorological parameters and scrub typhus incidence and its correlation analysis

The distribution of monthly meteorological parameters and the ST incidence were expressed as mean, standard deviation, median and interquartile range (IQR) at the monthly level (Supplementary Table S1). Autocorrelation and partial autocorrelation functions were preformed to assess seasonality and trend within the dependent variable itself. Cross-correlation function was used to examine lag-pattern relationships between meteorological variables and ST incidence time series. Spearman correlation analysis was performed on the monthly incidence of scrub typhus and same-month meteorological parameters from 2010 to 2019. Highly correlated meteorological variables were subsequently fit in separate models to explore the effects of meteorological parameters on the monthly incidence of scrub typhus.

#### 2.3.2 Distributed lag non-linear model

Analysis was conducted using distributed lag non-linear models (DLNM) using the “dlnm” and “ggplot2” packages in R version 4.3.0. DLNM models integrate concurrent non-linear and lag patterns, termed exposure-lag-response associations. These model are based on a “cross-basis” framework that utilizes a two-dimension space function to elucidate the dependencies between the predictors and long lags (24). In this study, the model was fitted with a quasi-Poisson distribution, and predictions were generated for a set of suitable predictor values and lag period values. The models allowed the relative risks (RR and [95% confidence interval]) at different levels of exposure variable and lag months to be estimated (24, 25).

The model was expressed as follows (24):


$$\begin{array}{l} \log\;E\left[Yt\right]=\alpha +cb\left(\mathrm{weather},\beta \right)+\sum \mathrm{ns}\left(Zi,df\right)\\ \;+\mathrm{ns}\left(\mathrm{time},df\right)+\mathrm{factor}\left(\mathrm{month}\right) \end{array}$$

Where E [Yt] is the expected number of monthly scrub typhus cases per 100,000 population on any given lag month, α is the intercept, $cb\left(\mathrm{weather},\beta \right)$ is the “cross-basis” function for transforming the main monthly variable and the incidence of scrub typhus detected over the lag period. The β parameters in “cross-basis” rely on basis functions describing smooth curves, such as splines or polynomial functions. The main monthly variable was based on the natural cubic spline function (ns) with various numbers of degrees of freedom (df) or of knots. Finally, the best fitting model for each main variable was selected having 3–5 degrees of freedom or 5 equally spaced knots. The lag was defined by a polynomial function with 2–3 degrees. The covariates were fitted via $\mathrm{ns}\left(Zi,df\right)$ with 3 to 8 df. Year was fitted via $\mathrm{ns}\left(\mathrm{time},df\right)$ with 3 df per year and month of the year as a factor to control the seasonality and long-term trend of models.

As monthly mean, maximum and minimum temperatures, and monthly temperature range were all highly correlated with each other (|r| > 0.8), these related temperature variables were fitted as the main exposure in separate DLNM models, with relative humidity and precipitation variables considered as covariates. When relative humidity served as the primary variable, precipitation and mean temperature were considered as covariates. Similarly, for the precipitation-focused model, relative humidity and mean temperature were the covariates. The robustness of these models was verified by adjusting the lag values and df of the basis function in the main variable, and adjusting df in covariates. When a wide range of relative humidity and precipitation was the main variable in separate models, the adjustment df were replaced with a natural cubic spline with equally spaced knots on the exposure-response association in the “cross-basis” function. Finally the lag months was set at 3 based on both a small quasi-Bayesian information criterion (QBIC) in the multiple-variable DLNMs and the incubation of the cycle of mites (average duration 2–3 months) (6, 26). The parameters of the final fitted models are shown in Table 1. The prediction values were compared with a reference defined as the midpoint of each meteorological parameter range. Three-dimension plots, contour and slice plots showing the lag-specific RRs for selected exposure levels, as well as plots of cumulative RR over each lag duration for selected exposure levels and plots of overall cumulative RR for the lag 0–3 period against selected exposure levels, were constructed based on the “crosspred” function. The goodness of fit was assessed through residual variation plots (27). Both lag-specific and cumulative RRs were estimated together with their 95% confidence intervals. A *p* value less than 0.05 was considered statistically significant.

**Table 1:** The parameters of DLNMs in the fitted models.

Main variable	Reference	Lag (month)	Character dimension^*^	Knots^*^	Covariates
Mean temperature (°C)	15°C	3	ns, df = 3	–	Relative humidity (df = 4), precipitation (df = 7)
Maximum temperature (°C)	26°C	3	ns, df = 4	–	Relative humidity (df = 4), precipitation (df = 7)
Minimum temperature (°C)	9°C	3	ns, df = 3	–	Relative humidity (df = 4), precipitation (df = 7)
Temperature range (°C)	17°C	3	ns, df = 5	–	Relative humidity (df = 3), precipitation (df = 3)
Relative humidity (%)	68%	3	ns	equalknots (df = 5)	Precipitation (df = 3), mean temperature (df = 3)
Precipitation (cm)	25 cm	3	ns	equalknots (df = 5)	Relative humidity (df = 5), mean temperature (df = 5)

^*^ns, natural cubic spline; df, degrees of freedom; equalknots, a natural cubic spline with equally spaced knots of the main meteorological parameter.

### 2.4 Ethical approval

The study was approved the Medical Ethics Committee of Dali University (No. MECDU-201901-3). The disease surveillance data used in this study were obtained from the CNDSS with approval from the Chinese Center for Disease Control and Prevention. All data obtained were anonymized without personal information.

## 3 Results

### 3.1 Monthly distribution of meteorological parameters and scrub typhus incidence

**Figure 2:**
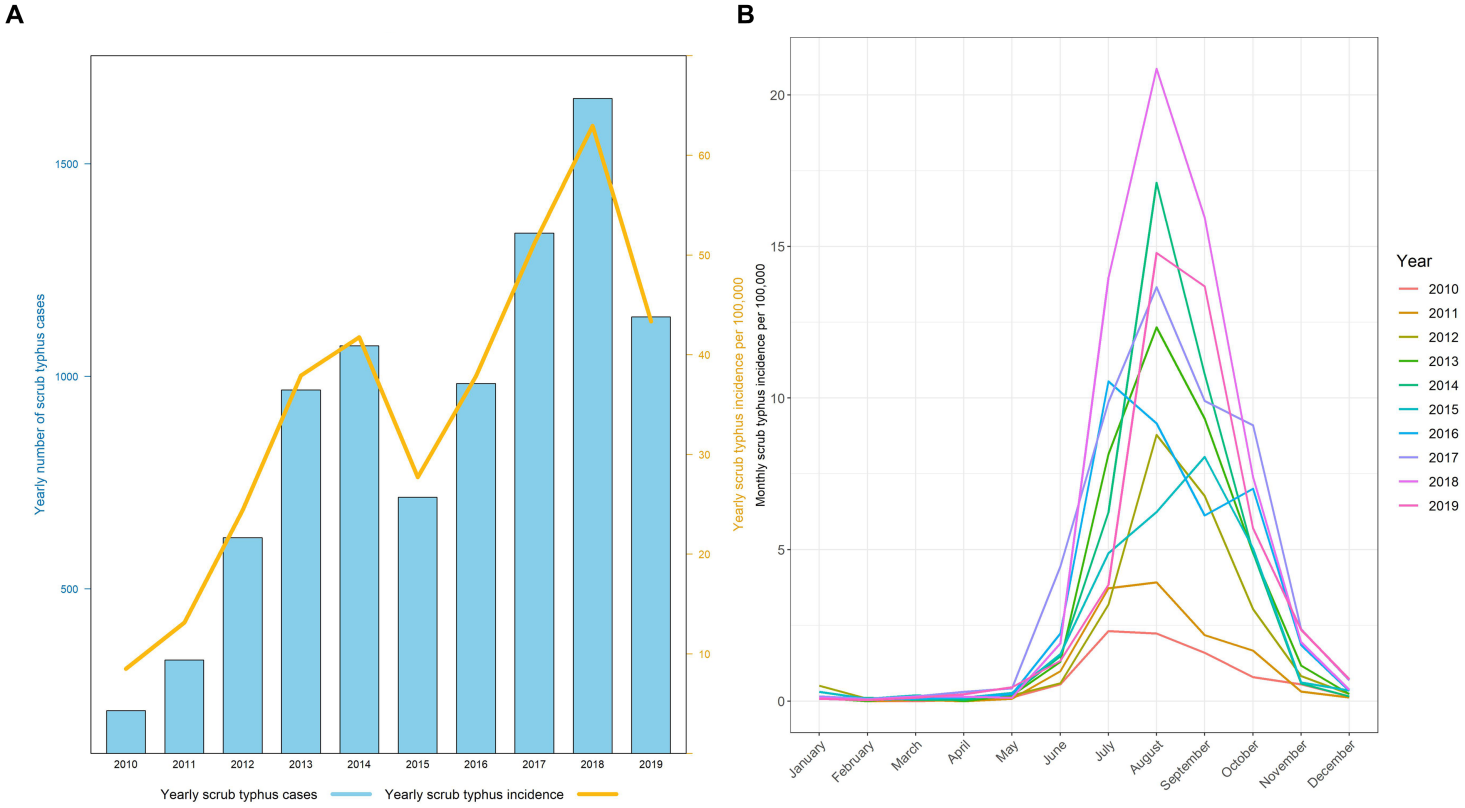
**(A)** Distribution of the yearly number cases and incidence of scrub typhus in Baoshan City from 2010 to 2019. **(B)** Monthly incidence of scrub typhus across different year.

A total of 9,034 scrub typhus cases was reported between 1st January 2010 to 31st December 2019 in Baoshan City, accounting for 21.86% (9,034/41,324) of the total scrub typhus cases in Yunnan Province. Overall, the annual scrub typhus incidence in Baoshan City increased from 8.49 per 100,000 in 2010 to 62.96 per 100,000 in 2018, with a dip in the trend in 2015 followed by a further increase. Year 2019 recorded a second drop in incidence, to 43.35 per 100,000 (Figure 2A). In each year, the monthly incidence was elevated from June to October, peaking in August. For the remaining months of the year, the numbers were minimal (Figure 2B).

**Figure 3:**
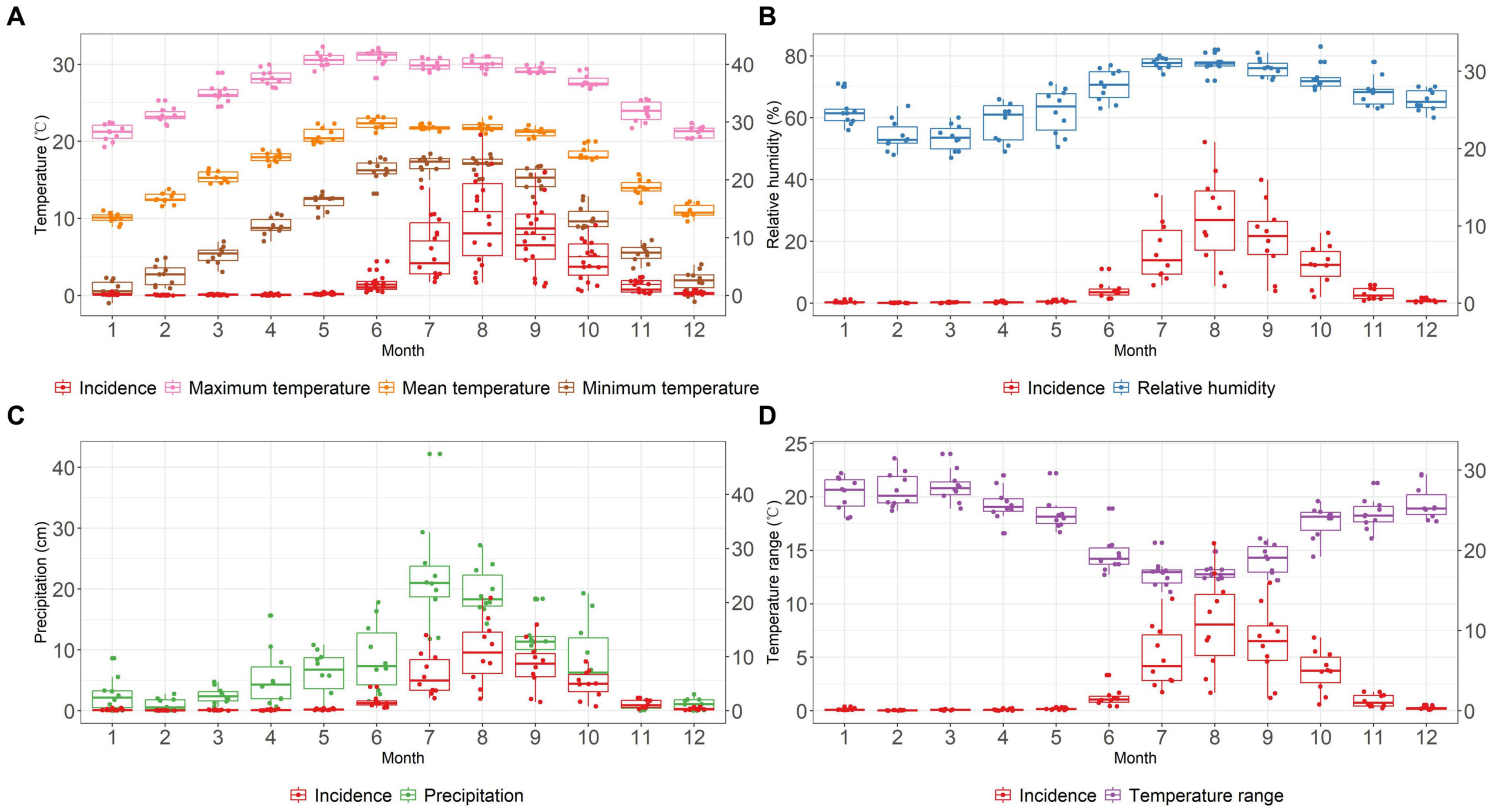
The time series of meteorological parameters and the incidence of scrub typhus at monthly level in Baoshan City, 2010–2019, from panels **(A–D)** plot, the red boxplot represents the incidence of scrub typhus, the *X* axis represents the months from January to December, the right-hand *Y* axis represents the monthly incidence of scrub typhus per 100,000. **(A)** Mean temperature (°C): orange boxplot, Maximum temperature (°C): pink boxplot, Minimum temperature (°C): brown boxplot. **(B)** Relative humidity (%): blue boxplot. **(C)** Precipitation (cm): green boxplot. **(D)** Temperature range (°C): purple boxplot.

In each year, before the increase of scrub typhus incidence in June, there was a gradual rise in monthly mean temperature, maximum temperature and minimum temperature (Figure 3A). The mean temperature peaked in June coinciding with the onset of a rise in scrub typhus incidence. During the period of rapidly increasing incidence, the year-to-year fluctuation of each monthly temperature parameter remained relatively stable throughout the 10 years of recording. However, as displayed in the boxplots in Figure 3D, there was a narrowing of year-to-year fluctuation in monthly temperature range during these months. Concurrently with the increasing and decreasing trend in scrub typhus incidence, the relative humidity and precipitation showed upward and downward trends, respectively (Figures 3B,C). Year-to-year fluctuation in humidity decreased at higher levels, while the fluctuation in precipitation was narrower at lower levels.

### 3.2 Correlation analysis between the monthly incidence of scrub typhus and concurrent meteorological parameters

Monthly scrub typhus incidence was positively correlated with each meteorological parameter (*p* < 0.001), except for monthly temperature range, with which the incidence was negatively correlated. Similarly positive intercorrelations were seen for all meteorological parameters, except for negative correlations with monthly temperature range (*p* < 0.001) (Supplementary Figure S1).

### 3.3 Impact of separate meteorological variables on the incidence of scrub typhus

#### 3.3.1 Lag-specific relative risk of meteorological parameters on the ST incidence

**Figure 4:**
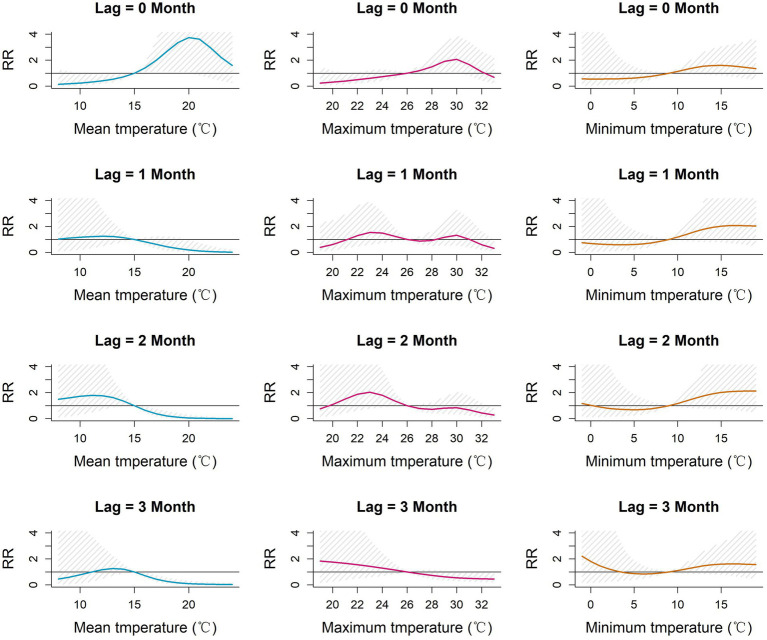
Lag-specific relative risk for mean, maximum and minimum monthly temperatures referenced to the mid-range of each parameter at each of lags 0–3 months in Baoshan City, 2010–2019. RR: Relative risk of the occurrence of scrub typhus referenced to the midpoint of each meteorological parameter range.

**Figure 5:**
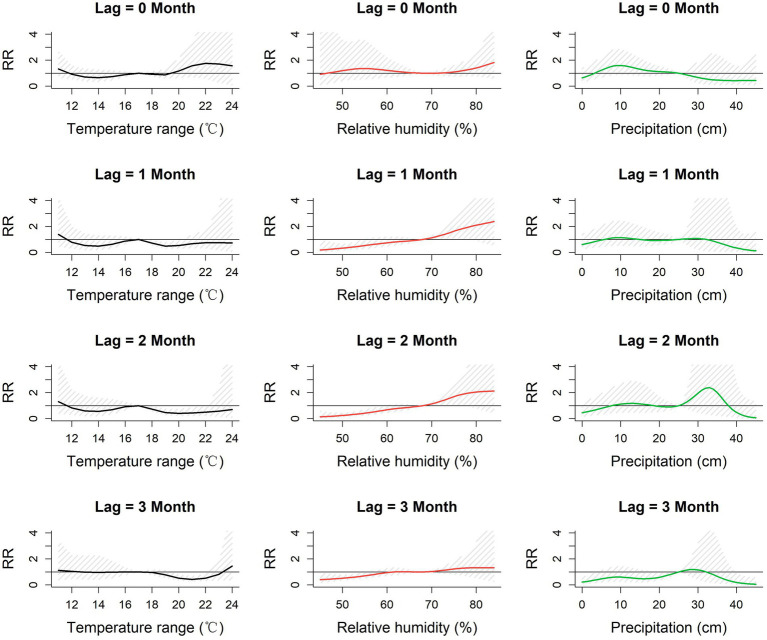
Lag-specific relative risk for monthly temperature range, relative humidity and precipitation referenced to the mid-range of each parameter at each of lags 0–3 months in Baoshan City, 2010–2019. RR: Relative risk of the occurrence of scrub typhus referenced to the midpoint of each meteorological parameter range.

The 3-dimensional and contour surfaces representing the lag-specific relative risk of each meteorological parameter derived from the DLNM models are shown in Supplementary Figures S2, S3. For each parameter, the midpoint of the parameter range was chosen as the reference value for estimation of RR. Slices from these surfaces, depicting the relative risk (RR) at specific lags for the association between monthly meteorological parameters and monthly incidence of scrub typhus in Baoshan City, are shown in Figures 4, 5. The highest RR of each meteorological parameter at each lag month is reported in Table 2. The RR of mean temperature in the current month (lag 0) increased with increasing mean temperature up to a peak of 3.74 [1.01–13.85] (ref 15°C) at 20°C. Similarly, the RR associated with maximum temperature in the current month increased to a peak of 2.07 [1.10–3.90] (ref 26°C) at 30°C. By contrast, in each of the subsequent 3 months (lags 1, 2 and 3), higher levels of mean and maximum temperature were associated with reduced lag-specific RRs.

**Table 2:** The highest lag-specific relative risks of scrub typhus by lag month for meteorological parameters in Baoshan City, western Yunnan Province, China, 2010–2019.^*^

Lag (month)	Mean temperature (°C)	RR (95%CI)	Maximum temperature (°C)	RR (95% CI)	Minimum temperature (°C)	RR (95%CI)	Temperature range (°C)	RR (95%CI)	Relative humidity (%)	RR (95%CI)	Precipitation (cm)	RR (95%CI)
		(Ref. = 15°C)		(Ref. = 26°C)		(Ref. = 9°C)		(Ref. = 17°C)		(Ref. = 68%)		(Ref. = 25 cm)
0	20	3.74	30	2.07	15	1.6	22	1.75	83	1.7	10	1.59
		(1.01, 13.85)		(1.10, 3.90)		(0.84, 3.04)		(0.60, 5.11)		(0.64, 4.54)		(0.89, 2.84)
1	12	1.24	23	1.54	17	2.07	16	0.88	83	2.3	10	1.13
		(0.47, 3.27)		(0.60, 3.90)		(0.68, 6.31)		(0.64, 1.21)		(0.62, 8.48)		(0.53, 2.43)
2	11	1.79	23	2.04	18	2.13	16	0.91	83	2.12	33	2.39
		(0.48, 6.71)		(0.79, 5.25)		(0.57, 7.99)		(0.64, 1.29)		(0.50, 8.89)		(0.30, 19.36)
3	13	1.26	20	1.75	16	1.62	24	1.45	81	1.33	29	1.18
		(0.72, 2.22)		(0.27, 11.54)		(0.73, 3.56)		(0.37, 5.72)		(0.45, 3.89)		(0.54, 2.60)

^*^RR, Relative risk of the occurrence of scrub typhus referenced to the midpoint of each meteorological parameter range.

The pattern of lag-specific RRs associated with minimum temperature across lags and temperature levels differed from those of the mean and maximum temperatures in that over the greater part of the minimum temperature range the RR increased with increasing temperature at all lags (maximum RR between 1.60 and 2.13 (ref 9°C), not statistically significant), though with slight and non-significant increases at the lowest values. Monthly temperature range showed slight but non-significant fluctuations of RR in the current month but marginally significant reduced lag-specific RRs (0.5–0.7, ref. 17°C) at temperature ranges of around 19–22°C at lags 1, 2 and 3.

Higher levels of relative humidity (above around 70%) were accompanied by increasing lag-specific RR at all lags, though most noticeably at lags 1 and 2. The lag-specific RRs (ref 68%) reached a maximum of 2.30 [0.62, 8.48] with a 1-month lag, and 2.12 [0.50–8.89] with a 2-month lag at 83%. The lag-specific RR associated with precipitation fluctuated throughout its range at all lags, but with a slight but non-significant peak of 2.39 [0.30, 1.4] (ref 25 cm) at lag 2 with a precipitation of 33 cm.

#### 3.3.2 Cumulative risk of meteorological parameters on the ST incidence

**Figure 6:**
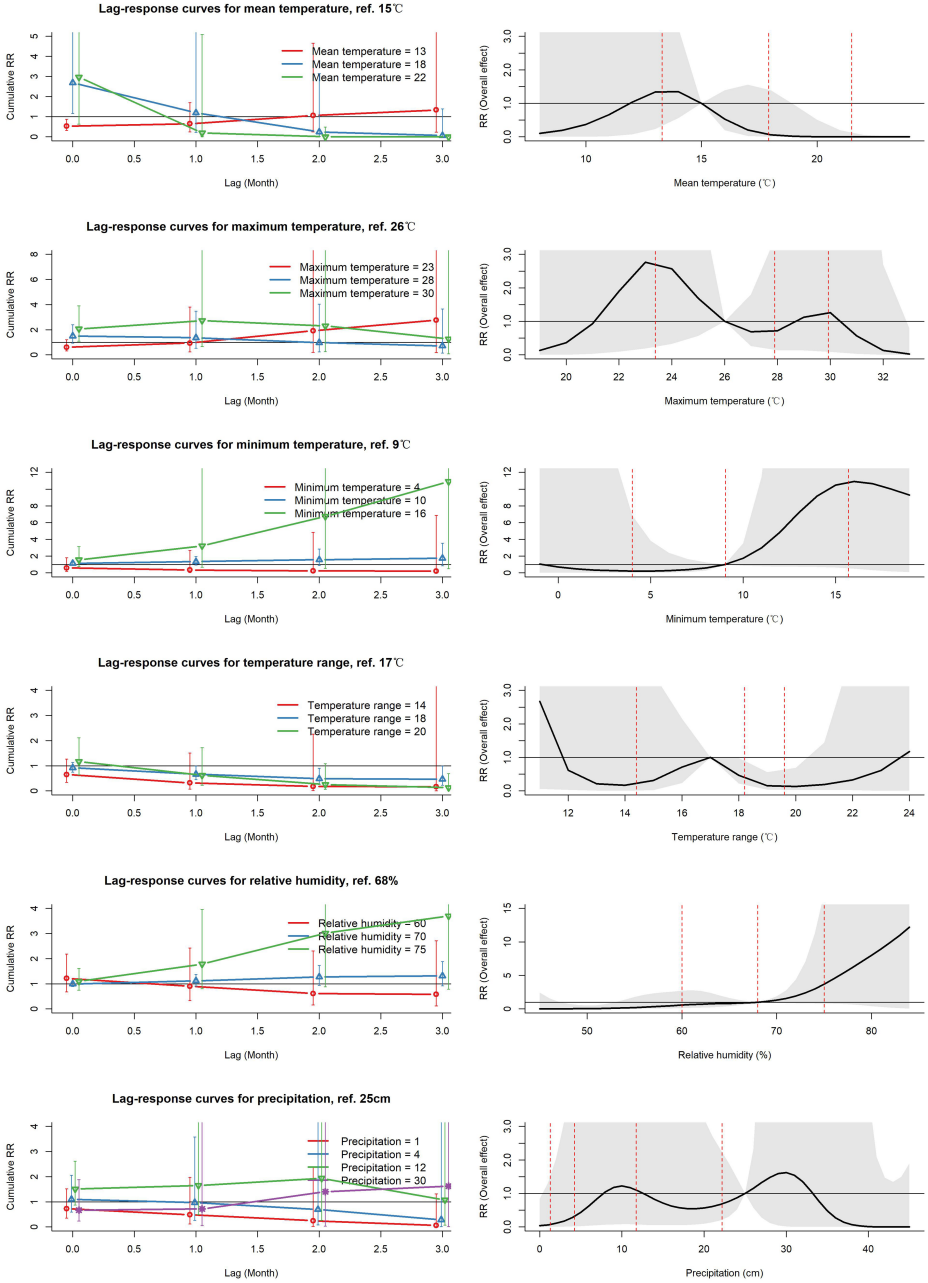
Left column: Cumulative relative risk of ST for each meteorological parameter over different lag periods from 0 to 3 at selected exposure levels of each parameter (representing the 25th, 50th and 75th percentiles). Right column: Cumulative (overall) relative risk of ST over the period from lags 0–3 according to the parameter value at the index month. Vertical lines in A and the shaded area in B represent the 95%CI of the cumulative risk referenced to t the midpoint of each meteorological parameter range. RR: Relative risk of the occurrence of scrub typhus referenced to the midpoint of each meteorological parameter range.

Based on these lag-specific RR profiles, the corresponding cumulative relative risk of the periods from the current month (lag 0) to lag 1, lag 2 and lag 3 at selected exposure levels the index month (lag 0) are shown in the left hand column of Figure 6, together with the lag 0 to lag 3 cumulative (overall) RR at different values of each parameter on the right hand column. The selected exposure levels included the 25th, 50th and 75th percentile values for each parameter with the addition of the 95th percentile for precipitation, for which the midpoint of the range coincided with the median value.

Whereas a high mean temperature of 22°C was associated with a higher incidence in the current month, the cumulative risk over the index and all subsequent months quickly declined. Meanwhile, a low value of mean temperature, as well as that of maximum temperature, showed slightly increasing cumulative risks over the ensuing months. However, the most clearly seen increases in cumulative risk over increasing lag periods were those for high minimum temperature and for high relative humidity (Figure 6 left column).

The lags 0–3 cumulative (overall) relative risk as a function of each parameter in the index month are displayed in the right hand column of Figure 6, and the value of each parameter associated with maximum overall risk indicated in Table 3. High values of cumulative relative risk (compared to the midpoint reference) were estimated for minimum temperature of 16°C (overall RR 10.9 [0.5, 237.54], ref. 9°C) and for relative humidity of 81% (overall RR 8.95 [0.3, 273.24], ref. 68%). Peak values with moderate overall relative risk for other parameters were lower: maximum temperature of 23°C (overall RR 2.76 [0.19, 39.87], ref. 26°C), precipitation of 30 cm (overall RR 1.62 [0.18, 147.88], ref. 25 cm), and mean temperature of 14°C (overall RR 1.35 [0.56, 3.25], ref. 15°C).

**Table 3:** The overall cumulative relative risk of meteorological parameters on the scrub typhus incidence over 0–3 month lag in Baoshan City, western Yunnan Province, China, 2010–2019.^*^

Meteorological parameters	Reference	The value at the highest overall cumulative RR	RR (95%CI)
			(Overall: 0–3 months lag)
Mean temperature (°C)	15°C	14°C	1.35 (0.56, 3.25)
Maximum temperature (°C)	26°C	23°C	2.76 (0.19, 39.87)
Minimum temperature (°C)	9°C	16°C	10.90 (0.50, 237.54)
Temperature range (°C)	17°C	23°C	0.61 (0.01, 59.30)
Relative humidity (%)	68%	81%	8.95 (0.29, 273.24)
Precipitation (cm)	25 cm	30 cm	1.62 (0.18, 147.88)

^*^RR, Relative risk of the occurrence of scrub typhus referenced to the midpoint of each meteorological parameter range.

## 4 Discussion

Apart from the overall increasing trend of scrub typhus incidence over the 10-year study period and the marked seasonal variation, non-linear associations between several monthly meteorological measures, particularly marked for minimum temperature and relative humidity, were shown to be associated with the risk of scrub typhus among the local population of Baoshan City over the current and/or subsequent 3 months. High mean and maximum temperatures were associated with a higher risk of scrub typhus in the current month respectively, while higher minimum temperature and higher relative humidity were followed by increasing cumulative risks over the ensuing 3 months. Higher precipitation was followed by increasing cumulative risk over the ensuing 2-month period. Compared with a moderate monthly maximum-minimum temperature range (17°C) both wider and narrower ranges were followed by a lower cumulative risk of scrub typhus over the ensuing 3 months.

Considering the predicted cumulative risk of scrub typhus over the ensuing 4-month period (0 to 3 month lags), the risk peaked following a moderate-to-low mean and maximum temperatures, (14°C and 23°C, respectively, around the 25th percentile level), high minimum temperature (16°C, around the 75th percentile), and moderate to high precipitation (10 cm, around the 75th percentile), though possibly also peaking following precipitation levels of around 30 cm. Increasing cumulative risk was seen for relative humidity increasingly above median levels (70%). Each of these conditions typically occurred in the months of March to June, depending on the year, thus the following 4 months included the usual months of high scrub typhus incidence. Meteorological parameters are crucial factors affecting the development and reproduction of mites (the vector and host of *Orientia tsutsugamushi*), which display heightened sensitivity to meteorological changes, particularly in temperature and humidity (28, 29). All species of mites complete the whole life cycle around in 2–3 months. Nonetheless, changes in meteorological variables can exert their influence on the occurrence and spread of scrub typhus through their impact on the life cycle of mites (6, 30). This study suggests that the occurrence of scrub typhus in any one current month may be linked to the hatching and developing of mites in the previous months, leading to certain lag associations between the meteorological parameters and the occurrence of scrub typhus. These findings provide valuable insights into understanding the mechanisms driving the high occurrence of scrub typhus in tropical and subtropical regions with complex environments and diverse animal populations. Previous studies conducted in Asia (such as South Korea, Japan and China) have similarly shown that the occurrence of scrub typhus is affected by meteorological variables, including temperature, relative humidity, precipitation, snowfall, wind speed and sunshine (15, 28, 31). However, these studies are insufficient for a comprehensive quantitative analysis of the lag associations between the meteorological parameters and scrub typhus incidence in the known endemic areas. In this study, the distributed lag non-linear model showed quantitative associations between monthly temperature-related variables, relative humidity, precipitation and scrub typhus incidence, providing an early warning signal before the peak month for the occurrence of scrub typhus to local authorities and generating scientific clues for the further research in similar environments.

Global warming in recent decades has an impact on vector-borne disease. Minor climate changes could lead to dramatic changes in the reproduction and carriage of pathogens, indirectly affecting the transmission of *Orientia tsutsugamushi* (32). Warm climates and wet habitats can be valuable predictors to assess the outbreak risk of scrub typhus, as they directly facilitate the development cycle of mites, the hatching and climbing speed of mites, and the abundance and distribution of small mammals. In addition, appropriate climate conditions often prompt outdoor activities by humans, and elevate the possibility of contact with infected mites or small mammals in nature (29, 30, 33, 34). In general, mites have seven stages through the whole life cycle, namely egg, deutovum, larva, nymphochrysalis (nymphophane or protonymph), nymph, imagochrysalis (tritonymph) and adult. Each stage has different optimal temperature for development and reproduction (6). Previous studies conducted in laboratory conditions have shown that *L. deliense*, one of the dominant species in Yunnan Province, demonstrates adaptability for survival and reproduction within the temperature range of 12–36°C (30, 35). The mites exhibit activity when the temperature exceeds 10°C and attempt to crawl when it surpasses 12°C (6, 35). Female mites mainly deposit their eggs from March to May, with egg-laying rates decreasing when the temperature ranges rise between 25 and 30°C (30). The optimal temperature for larva hatching and development is above 22°C, and temperatures between 18 and 30°C are conducive for mites to seek hosts for pathogen transmission (21, 30, 35, 36). Adult mites remain in the soil for at least 15 months, while the larva stage can survive several months until the appropriate temperature for feeding (29). In this study, moderate-to-low mean and maximum temperatures, (13°C and 23°C, respectively, around the 25th percentile level), were more likely reported from February to March in Baoshan City, which is suitable for mites to lay the eggs, hence the effect on the spread of scrub typhus becoming apparent after a lag of several months. Conversely, when the temperature reaches 20°C to 30°C (high mean and maximum temperatures), observed from June to September in Baoshan City, it becomes the most suitable period for mites to seek hosts to feed on. Moreover, scrub typhus infections are not recognized immediately after a bite; both animal hosts and human shows symptoms within 6 to 21 days after being bitten (37). Therefore, the effect of risk of monthly mean and maximum temperature, at 20°C and 30°C, respectively, could be observed in the current month or within 30 days. In addition, this study also indicated that increased monthly minimum temperature could lead to high risk of the occurrence of scrub typhus. This result is consistent with previous study showing that mite activity decreases when the temperature drops below 10°C (21). In southern Yunnan, *L. deliense* is the summer-autumn type, with the highest numbers in July (38). Assuming the temperature increases before the summer season, it could create an appropriate environment for mites to deposit the eggs and develop to the larva stage. This progression increases the abundance of *L.deliense* and indirectly increases the probability of carriage and transmission of *Orientia tsutsugamushi* in mites. Furthermore, the elevated temperature may foster a more suitable habitat for other mite species, such as *L.rubellum* and *L.sialkotense*, recognized vectors of *Ot* and recently found coexist in the southern region of Yunnan Province (23, 39), which could increase the probability of the transmission of *Ot* in natural environments. As a result, long-term monitoring of temperature changes and the abundance of mites may provide early warning before disease occurs in human. When temperatures rise, signaling a potential disease risk, proactive measures should be taken to control mite abundance and minimize the density of small mammal during the mite’s development period. During increasing periods of scrub typhus cases, prioritizing human intervention becomes imperative, emphasizing health education initiatives and implementing physical protective measures to mitigate the spread of the disease.

The cumulative risk of scrub typhus was increased over the ensuing 3 months when the relative humidity increased above the median levels (around 70%), which is consistent with previous studies showing that 60–95% relative humidity was the optimum for the occurrence of scrub typhus in other regions of China (22, 30). One previous study showed that mites (*L. deliense*) given a certain temperature (25°C) and placed in different humidities (20–100%) could survive longer in higher humidity (35). The higher humidity provides an ideal environment for mite survival and reproduction. Moreover, humid conditions are conducive to vegetation growth based on the moisture of the soil, providing a suitable shelter for small mammals, which in turn contributes to pathogen’s preservation and transmission in nature (1, 15, 22, 26). For precipitation, the cumulative risk of scrub typhus was separately peaked at precipitation level of 10 cm and 30 cm. The effect of precipitation on scrub typhus can be explained by the increase in vegetation as precipitation increases, which makes survival and reproduction easier for small mammals and results in a high density of small mammals in nature (22, 40).

The study has some limitations. First, previous studies have used weekly meteorological data, whereas in this study was based on monthly data; however, we added the monthly maximum and minimum temperatures and monthly temperature range to examine in more detail the possible effects of temperature for the occurrence risk of scrub typhus from multiple perspectives. Second, the study addressed the factors influencing scrub typhus incidence in the known high endemic area in western Yunnan. This area includes two main cities reported the number of scrub typhus, Baoshan City and Lincang City, but our analysis was confined to only the former. Climatic differences between these two cities, partly influenced by their different topography, suggest that the associations revealed in our study may not fully represent the situation in the whole of western Yunnan. Third, the scrub typhus cases were identified from passive surveillance data recorded in the China CDC system; some cases who did not enter the health system may be missing from our data.

## 5 Conclusion

A lag non-linear association was observed between monthly meteorological parameters (temperature-related variables, relative humidity and precipitation) and scrub typhus incidence in Baoshan City. Warmer monthly temperatures facilitated the occurrence of scrub typhus in the current month. In contrast, low monthly mean and maximum temperatures were more likely to produce an increase in scrub typhus cases occurring several months later. Increasing monthly minimum temperature and relative humidity could be linked to the higher cumulative risk of scrub typhus over the ensuing 3 months.

## Data availability statement

The original contributions presented in the study are included in the article/Supplementary material, further inquiries can be directed to the corresponding authors.

## Ethics statement

The studies involving humans were approved by the Medical Ethics Committee of Dali University (No. MECDU-201901-3). The disease surveillance data used in this study were obtained from the CNDSS with approval from the Chinese Center for Disease Control and Prevention. All data obtained were anonymized without personal information. The studies were conducted in accordance with the local legislation and institutional requirements. Written informed consent for participation was not required from the participants or the participants’ legal guardians/next of kin in accordance with the national legislation and institutional requirements.

## Author contributions

Y-YL: Conceptualization, Data curation, Formal analysis, Investigation, Methodology, Project administration, Software, Validation, Visualization, Writing – original draft, Writing – review & editing. AG: Conceptualization, Formal analysis, Investigation, Methodology, Software, Supervision, Validation, Visualization, Writing – original draft, Writing – review & editing. J-XY: Conceptualization, Data curation, Funding acquisition, Methodology, Project administration, Resources, Supervision, Validation, Writing – review & editing.

## Footnotes

^1^ http://www.chinacdc.cn/

^2^ http://www.chinacdc.cn/tzgg/200901/t20090105_40316.html

^3^ http://stats.yn.gov.cn/

^4^ http://data.cma.gov.cn/
