# Supplementary material for: The impact of meteorological parameters on the scrub typhus incidence in Baoshan City, western Yunnan, China
Source: Front Public Health. 2024 Apr 24;12:1384308. doi: 10.3389/fpubh.2024.1384308 (PMC11078032; doi:10.3389/fpubh.2024.1384308)
Supplement: Supplementary file 1 [file Data_Sheet_1.docx]

**Supplementary Materials**

Table S1. Summary for monthly scrub typhus incidence and meteorological parameters in Baoshan City, 2010-2019.

| Variables (Monthly) | Mean | SD | Min. | P25 | P50 | P75 | Max. |
| --- | --- | --- | --- | --- | --- | --- | --- |
| Scrub typhus incidence per 100,000 | 2.91 | 4.43 | 0.00 | 0.12 | 0.56 | 4.05 | 20.86 |
| Mean temperature (℃) | 17.27 | 4.39 | 8.90 | 13.30 | 17.90 | 21.50 | 23.20 |
| Maximum temperature (℃) | 26.89 | 3.61 | 19.30 | 23.38 | 27.90 | 29.93 | 32.30 |
| Minimum temperature (℃) | 9.41 | 6.02 | -1.00 | 4.00 | 9.05 | 15.70 | 18.40 |
| Temperature range (℃) | 17.48 | 3.23 | 11.10 | 14.40 | 18.20 | 19.60 | 24.00 |
| Relative humidity (%) | 66.60 | 9.41 | 47.00 | 60.00 | 68.00 | 75.00 | 83.00 |
| Precipitation (cm) | 7.56 | 8.04 | 0.00 | 1.28 | 4.22 | 11.72 | 42.18 |


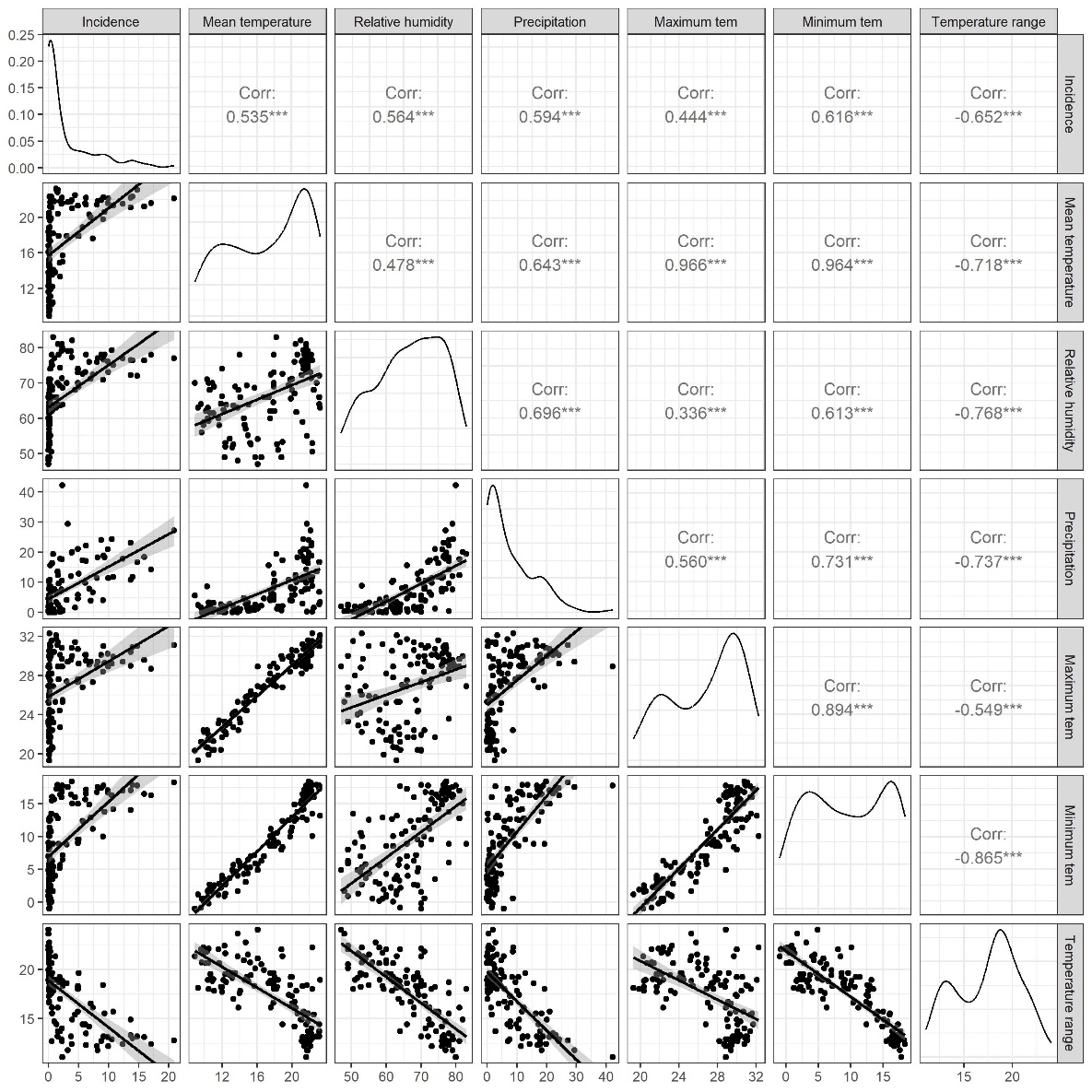


Figure S1. Spearman’s correlation matrix of the scrub typhus incidence and meteorological parameters in Baoshan City, western Yunnan Province, China.


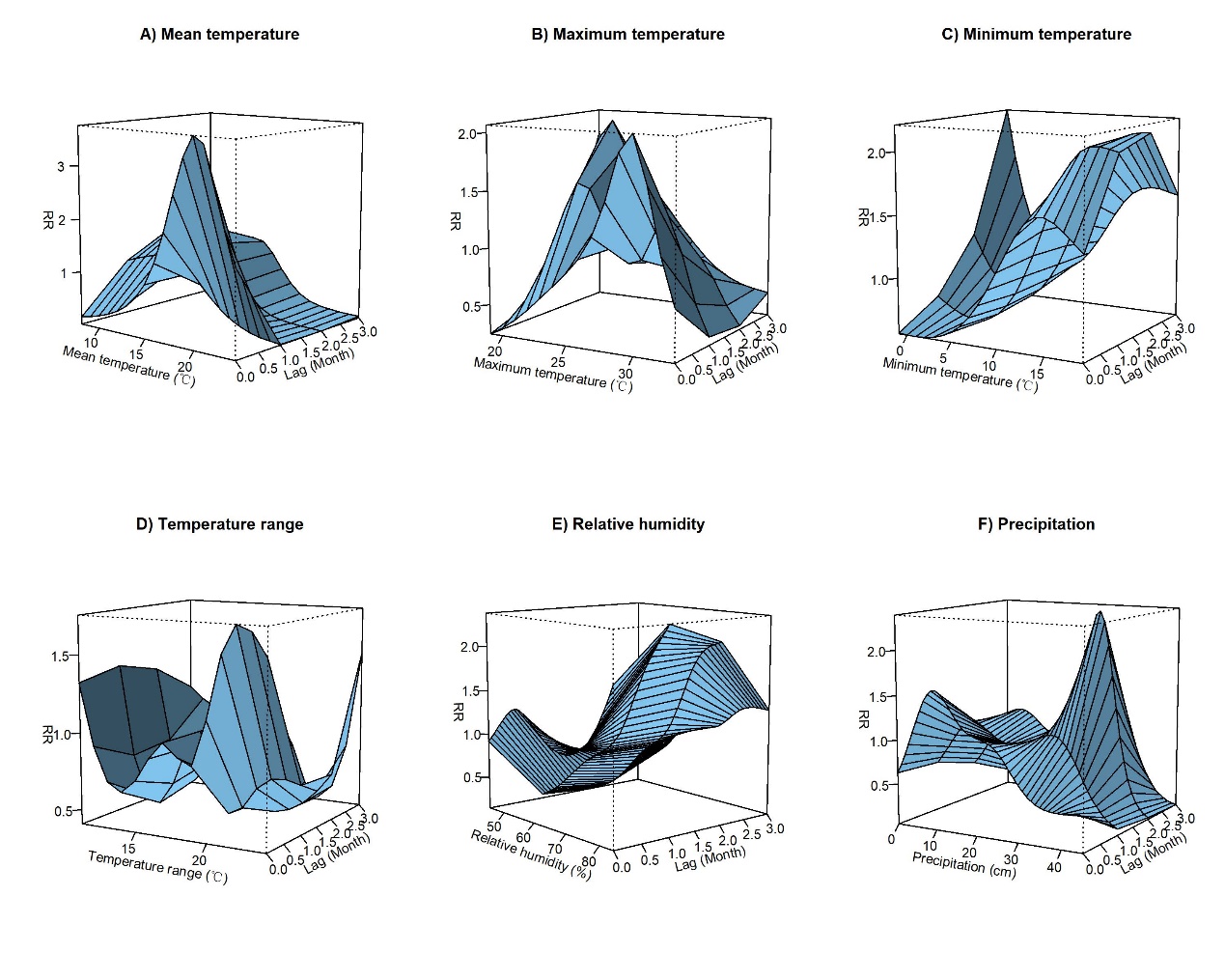


Figure S2. 3-D plots of the relative risk of meteorological parameters on the scrub typhus incidence, including (A) mean temperature, (B) maximum temperature, (C) minimum temperature, (D) temperature range, (E) relative humidity and (F) precipitation. The X-axis, Y-axis represented the various meteorological parameters and the month-lag, the Z-axis represented the effect of relative risk of each meteorological parameters on the scrub typhus incidence based on month-lag.


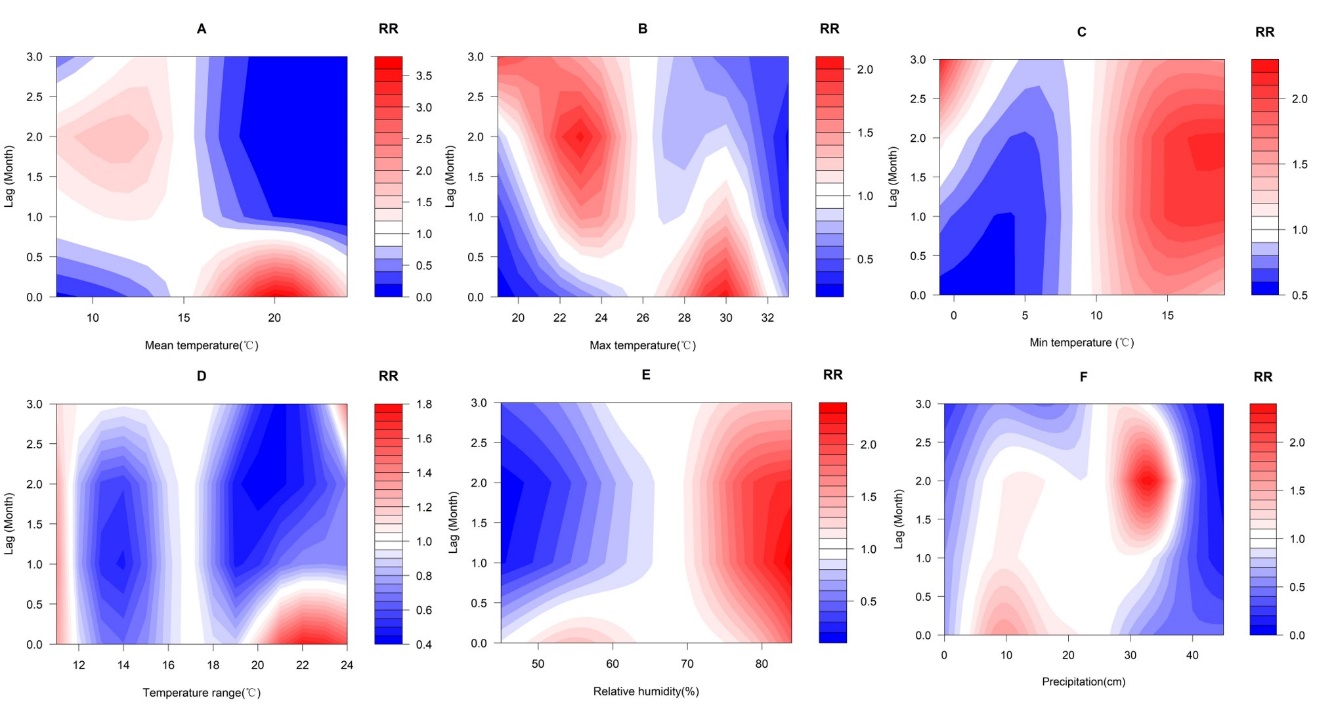


Figure S3. Contour plots of the relative risk of meteorological parameters on the scrub typhus incidence, including (A) mean temperature, (B) maximum temperature, (C) minimum temperature, (D) temperature range, (E) relative humidity and (F) precipitation.
